# Cellular and Molecular Mechanisms in Mycobacterial Infection

**DOI:** 10.3390/ijms23137205

**Published:** 2022-06-29

**Authors:** Natalie E. Nieuwenhuizen, Joanna C. Evans

**Affiliations:** 1 Institute for Hygiene and Microbiology, Julius-Maximilians-Universität Würzburg, 97080 Würzburg, Germany; 2 The Francis Crick Institute, 1 Midland Road, London NW1 1AT, UK

Tuberculosis (TB), caused by the bacillus *Mycobacterium tuberculosis (Mtb)*, remains a leading cause of death by infectious disease, overshadowed only recently by the COVID-19 pandemic. Key goals in the reduction of the global TB incidence include the improvement of socioeconomic conditions, efficacious vaccination, early diagnosis, and effective antitubercular chemotherapy, preferably with shorter treatment regimens. This Special Issue presents a collection of research articles and reviews covering aspects such as *Mtb* biology, the development of novel therapeutics, host immune responses targeting *Mtb*, and the development of novel vaccine candidates for the prevention of TB.

A major contributing factor to the success of *Mtb* as an ancient pathogen is its ability to cause asymptomatic latent infection, allowing it to persist undetected in the host for decades until a breach in the immune system enables its reactivation, thereby facilitating ongoing transmission. A significant challenge in the quest to reduce TB incidence is that bacilli in the latent state are thought to be largely non-replicating and/or metabolically inactive, making them recalcitrant to most available TB chemotherapeutics. Despite significant efforts to understand and define the physiological state of *Mtb* during latency, the complexity of the granulomas in which the bacilli reside makes in vitro recapitulation of the host environment exceptionally challenging. In this Special Issue, Campaniço and colleagues provide a comprehensive review of our current understanding of latency, wherein they discuss the successes and limitations associated with current therapies and diagnosis techniques, existing in vitro approaches to mimicking latency, and potential new targets and compounds in the drug discovery pipeline [1]. Their work highlights the significant gaps in our understanding of the complexity and development of the latent state, which urgently need to be filled in order to inform highly efficacious solutions for the treatment of TB.

In addition to the ability of *Mtb* to establish latent infection, the ongoing emergence of strains resistant to all available antimycobacterial agents further contributes to the continued success of the bacillus, and underscores the need to identify novel approaches for the treatment of TB disease. As integral components of the human innate immune response, antimicrobial peptides have been proposed as candidates to complement conventional drug therapy for TB treatment, but our knowledge of the cellular and molecular mode of action of these molecules is incomplete. In this Special Issue, Deshpande et al. exploit the superior resolution of confocal-based stimulated-emission depletion (STED) microscopy to gain further mechanistic insight into the interaction of the antimicrobial peptide LL-37 with *Mtb* [2]. Using this novel approach, the authors were able to visualise the internalisation of LL-37 by both *Mtb*-infected and uninfected primary human macrophages, as well as its localisation within the cell wall of internalised and extracellular bacilli. Their findings suggest that LL-37 exerts its antimycobacterial activity through the disruption of the cell wall, simultaneously providing further insight into the mechanism of action of LL-37 and validating the use of STED imaging for interrogating host–pathogen–peptide interactions. 

Although the global burden of mycobacterial disease is primarily attributable to *Mtb*, infections caused by nontuberculous mycobacteria (NTM) are steadily increasing globally. A member of the NTM complex, *M. abscessus* (*Mab*) is notoriously difficult to treat due to its intrinsic resistance to most available antibiotics. As such, the current standard of care is a regimen containing macrolides, even though the readily inducible resistance of *Mab* to this class of antibiotics often results in treatment failure. In an effort to identify novel classes of antibiotics for the treatment of *Mab* infection, Kim et al. describe the promising activity of epetraborole, which displayed greater efficacy in an in vivo zebrafish model than was observed with tigecycline [3]. Although epetraborole, which acts as a leucyl-tRNA synthetase inhibitor, entered phase II clinical trials for the treatment of TB in 2012, it was discontinued due to the rapid emergence of resistance. Despite this, however, the drug received fast track status from the US FDA for the treatment of NTM infections in January 2022, highlighting both the desperate requirement for novel therapeutics to treat NTM infection as well as the significant impact of this work by Kim and colleagues.

The elucidation of the sequence of the *Mtb* genome in 1998 facilitated significant advances in mycobacterial genomics, yet the functions of the majority of unique PE/PPE/PGRS proteins, which are found exclusively in mycobacteria and account for nearly 10% of the *Mtb* genome, remain poorly characterised. In this Issue, Sharma and colleagues provide a comprehensive review of the current literature describing the function(s) of PE/PPE/PGRS proteins [4], and also contribute to existing knowledge by demonstrating that the functionality of the PGRS domain of Rv0297 (PE_PGRS5) in *Mtb* virulence and pathogenesis is calcium-dependent [5]. Furthermore, they demonstrate that calcium enhances the interaction of Rv0297PGRS with surface-localised Toll-like receptor 4 of macrophages, leading to nitric oxide production and cytokine release, thus identifying a role for calcium-modulated PE-PGRS proteins in the virulence of *Mtb*, and suggesting that they could be exploited for therapeutic interventions.

As well as antibacterial approaches to treating TB, the development of host-directed therapies is currently being explored, for which a thorough understanding of the host immune response to *Mtb* is critical. For this Special Issue, Ruiz et al. reviewed the current literature on tumour necrosis factor (TNF), a cytokine that plays an important role in regulating innate and adaptive immune responses during mycobacterial infections [6]. TNF occurs in both transmembrane and soluble forms, and its multiple effector functions are dependent on the interactions of these forms with the TNF receptors (TNFR) 1 and 2. In their review, Ruiz et al. examine the impact of these interactions in the context of mycobacterial infections. Amongst other functions, the TNF/TNFR axis plays a critical role in the maintenance of a subpopulation of myeloid cells expressing CD3, which are increased around the caseous zone of granulomas and in the peripheral blood of patients with pulmonary TB, as well as in mice infected with BCG [7]. Recently, two subpopulations of CD3^+^ myeloid cells were discovered, one type expressing the T cell receptor (CD3^+^TCRαβ^+^) and the other not (CD3^+^TCRαβ^−^). Ramon-Luing and co-workers characterized the migration ability of three phenotypes of monocyte-derived macrophages (MDMs), CD3^−^, CD3^+^TCRαβ^+^, and CD3^+^TCRαβ^−^, upon infection with virulent (H37Rv) or avirulent (H37Ra) *Mtb* strains. H37Rv infection increased the frequency of CD3^+^ MDMs expressing TCRαβ compared to H37Ra, and decreased the migration ability of CD3^−^ MDMs, but not CD3^+^ MDMs. 

While macrophages are the primary host cells of *Mtb* bacilli, and are important in their killing, TB is also characterized by an influx of neutrophils into the lungs. While some beneficial roles have been ascribed to neutrophils [8], they are also important mediators of lung pathology and tissue destruction [8,9]. Borkute and colleagues have reviewed the interactions of neutrophils of *Mtb*, including the cell fate upon infection and intracellular events, as well as crosstalk between neutrophils and other cells [8]. They highlight the role of neutrophil in TB pathology, with a view to host-directed therapy. Along these lines, Linneman et al. evaluated myeloperoxidase (MPO), a peroxidase expressed predominantly by neutrophils, as a target for host-directed therapy in TB, using a mouse model [9]. This followed studies in human neutrophils showing that the inhibition of MPO prevented neutrophil necrosis and promoted apoptosis, improving mycobacterial killing by macrophages subsequently taking up the infected neutrophils. Importantly, this study revealed that, in contrast to human neutrophils, mouse neutrophils did not respond to *Mtb* by MPO production, an important inter-species difference that highlights the inadequacy of the mouse model in recapitulating certain features of TB, as discussed by Campaniço et al. [1]. 

Another important aspect of TB control is vaccination. The current TB vaccine in clinical use, Bacille Calmette–Guerin (BCG), has been a focus of much TB work in recent years. BCG has insufficient efficacy, and is considered unsuitable for use in immunocompromised individuals due to the risk of adverse events. Accordingly, much research has been carried out in recent years to develop an improved vaccine, as either a replacement or a booster for BCG. In this Special Issue, Belnoue et al. report the generation and testing of a recombinant LCMV (rLCMV) expressing the mycobacterial antigens TB10.4 and Ag85B, shared by BCG and *Mtb* [10]. As a standalone vaccine, rLCMV elicited high frequencies of polyfunctional *Mtb*-specific CD8 and CD4 T cell responses in mice and reduced lung *Mtb* burdens upon aerosol challenge, and when used as a booster to BCG vaccination, it augmented responses to the rLCMV-encoded antigens. However, protection after the rLCMV booster was not different from that provided by rLCMV or BCG alone. Nevertheless, the rLCMV vector appears to be a promising platform for the delivery of antigens when the elicitation of CD8 and CD4 T cells responses is required.

In summary, this Special Issue comprises a variety of articles that we hope will interest a broad audience and contribute towards the overall aim of tackling TB, at a time when deaths due to TB have risen for the first time in over a decade due to the COVID-19 pandemic. 
